# Invasive Non-groupable Meningococcal Disease Complicated by Brain Abscess Revealing C6 Complement Deficiency in a Young Adult

**DOI:** 10.7759/cureus.114004

**Published:** 2026-08-05

**Authors:** Guillermo Caceres-Cardenas, Ana Bove, Jennifer Ortega, Judith Berger

**Affiliations:** 1 Internal Medicine, St. Barnabas Hospital (SBH) Health System, Bronx, USA; 2 Infectious Diseases, St. Barnabas Hospital (SBH) Health System, Bronx, USA

**Keywords:** brain abscesses, complement deficiency, meningococcal meningitis, neisseria meningitidis, • septic shock

## Abstract

A 21-year-old man presented with sickle cell trait with septic shock, altered mental status, and respiratory failure. Blood cultures grew non-groupable *Neisseria meningitidis*. His course was complicated by consumptive coagulopathy, stress-induced cardiomyopathy, and a 2.8×1.2 cm rim-enhancing brain lesion concerning for abscess. Transesophageal echocardiography showed no endocarditis, supporting hematogenous seeding. The abscess resolved with prolonged intravenous ceftriaxone. Outpatient immunologic evaluation revealed low CH50 and C6 complement deficiency, prompting vaccinations and long-term penicillin prophylaxis. This case highlights brain abscess as a rare complication of meningococcal disease and underscores the importance of evaluating for complement deficiency in atypical presentations.

## Introduction

Invasive *Neisseria meningitidis* infection is a rapidly progressive, life-threatening disease that most commonly presents as meningitis or meningococcemia [1]. The incidence and clinical presentation vary with age, with meningitis and septicemia occurring most frequently in children and young adults, and the highest case fatality rate being observed in older adults, approaching 20% in individuals aged ≥60 years [2].

This pathogen is classified according to its polysaccharide capsule. Serogroups A, B, C, W, X and Y cause most meningococcal disease cases in the world, and the nongroupable or non-encapsulated meningococci rarely cause invasive disease, with cerebral abscess representing an exceptionally rare manifestation, and reported cases occurring mostly among people with complement deficiencies or receiving complement component inhibitor medications [3,4]. Despite advances in antimicrobial therapy and critical care, it remains associated with high morbidity and mortality, particularly when diagnosis or treatment is delayed [5].

*N. meningitidis* is an encapsulated Gram-negative diplococcus that colonizes the nasopharynx and is transmitted via respiratory droplets. While asymptomatic carriage is common, invasive disease occurs when the organism breaches mucosal barriers and enters the bloodstream. From there, it can disseminate hematogenously to cause meningitis, characterized by acute inflammation of the meninges, and meningococcemia, which may rapidly progress to septic shock and multiorgan failure. Several virulence factors, including its polysaccharide capsule and ability to evade complement-mediated killing, contribute to its pathogenicity. Host immune status plays a critical role in determining susceptibility and disease severity [1].

Complement plays a central role in host defense against *Neisseria* species, especially through the terminal pathway responsible for membrane attack complex formation [6]. Deficiencies in complement components (particularly C5-C9) significantly increase susceptibility to invasive meningococcal disease and are often associated with recurrent or unusually severe infections due to impaired bacterial clearance [7]. Recognition of these immunodeficiencies is important, as they have direct implications for secondary prevention, including vaccination and antibiotic prophylaxis [8].

Here, we report a case of a previously healthy young adult who presented with fulminant meningococcal sepsis complicated by brain abscess and multisystem involvement. Further evaluation ultimately revealed an underlying complement deficiency, underscoring the importance of immunologic workup in severe or atypical presentations of invasive *N. meningitidis* infection.

## Case presentation

A 21-year-old man with a history of sickle cell trait who immigrated from Guinea four months prior presented to our institution after a syncopal episode. On arrival, he was febrile, hypotensive, and altered, requiring endotracheal intubation for airway protection. He was found to be in septic shock and was started on empiric therapy for suspected meningitis with vancomycin, cefepime, and acyclovir. Blood cultures obtained at admission grew *N. meningitidis* (non-groupable), and antimicrobial therapy was subsequently narrowed to intravenous ceftriaxone.

Lumbar puncture demonstrated mild pleocytosis with negative cerebrospinal fluid cultures. Initial transthoracic echocardiography raised concern for mitral and tricuspid valve vegetations; however, transesophageal echocardiography showed no evidence of endocarditis. A reduced ejection fraction of 30%-35% on initial evaluation normalized on repeat imaging, consistent with stress-induced cardiomyopathy. The hospital course was further complicated by consumptive coagulopathy, which resolved with treatment of the underlying sepsis and supportive measures including fresh frozen plasma, platelets, and vitamin K.

**Figure 1:**
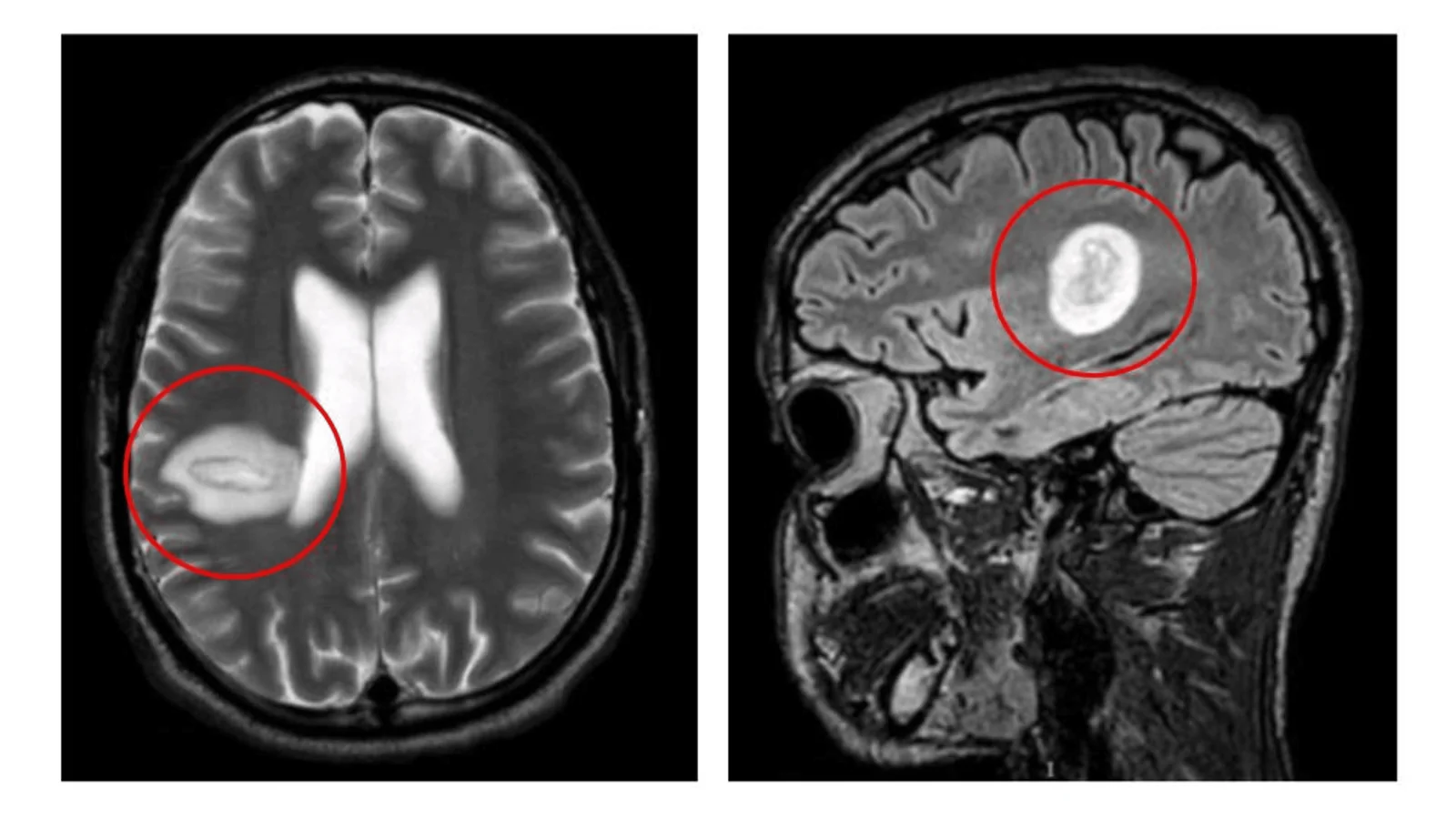
MRI brain with and without contrast showing a 2.8x1.2cm abscess in the right corona radiata

**Figure 2:**
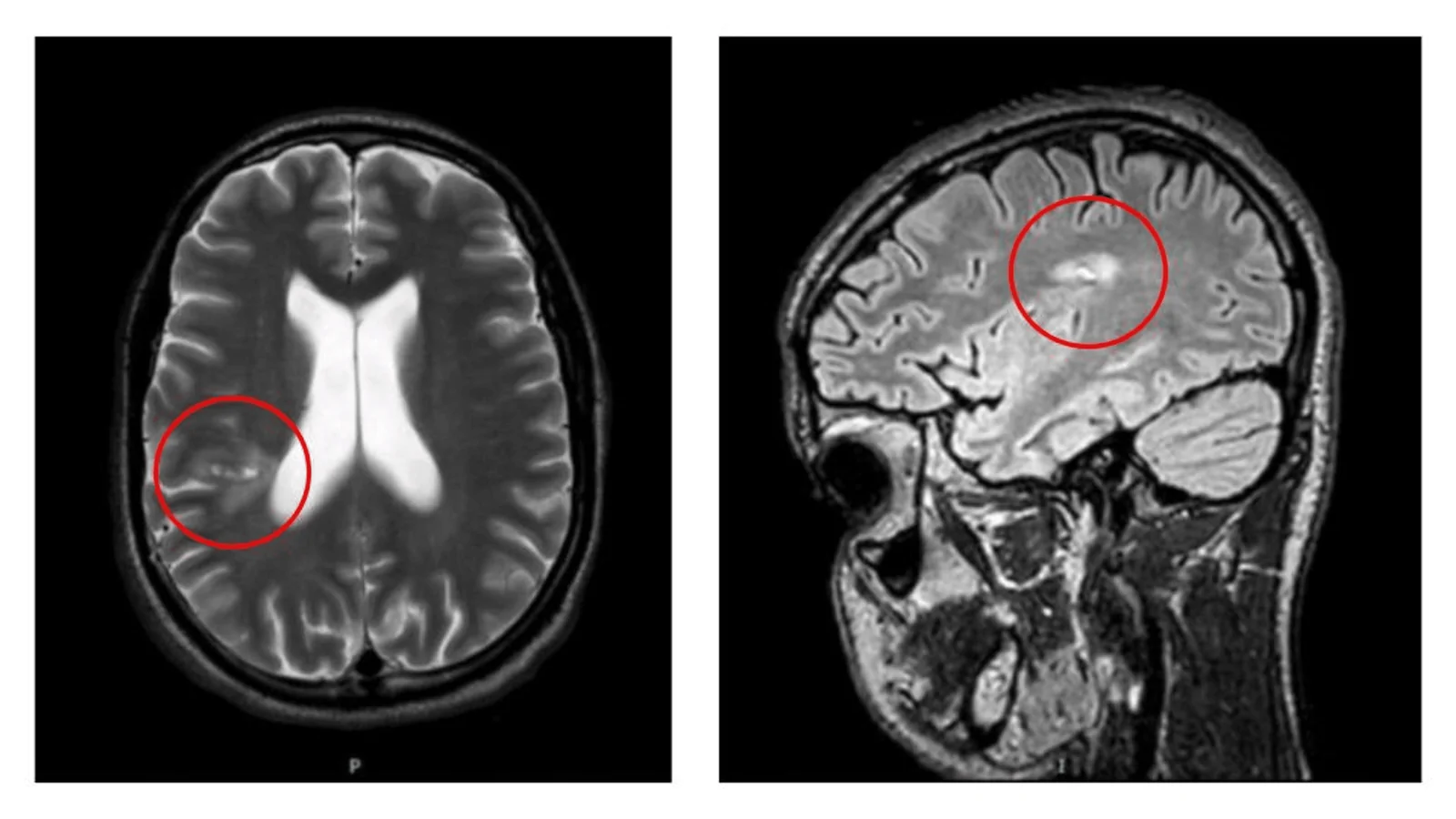
MRI brain with and without contrast after four weeks of antimicrobial therapy

Following extubation, the patient had persistent left upper extremity and bilateral lower extremity weakness. Repeat computed tomography of the head demonstrated new hypoattenuation, and subsequent magnetic resonance imaging revealed a 2.8×1.2 cm rim-enhancing lesion in the right corona radiata with central diffusion restriction, concerning for a brain abscess (Figure 1). Neurosurgical evaluation recommended conservative management, given the limited size of the lesion and clinical stability. Serial MRI studies obtained at two-week intervals demonstrated progressive reduction in lesion size, with near-complete resolution after a month (Figure 2). The patient completed an eight-week course of intravenous ceftriaxone with clinical improvement. Initial complement testing during hospitalization was within normal limits.

At outpatient follow-up, further immunologic evaluation revealed low total complement activity (CH50 <10 U/mL, range reference >41 U/mL) with deficiencies in C6 (<20 mcg/mL, range reference 28-69 U/mL), confirming complement deficiency. The patient received recommended vaccinations, including meningococcal, pneumococcal, *Haemophilus influenzae* type b, hepatitis, and human papillomavirus vaccines. After shared decision-making, he was initiated on monthly intramuscular penicillin for prophylaxis. Neurology attributed his mild residual left hand weakness to the prior brain abscess, with significant improvement achieved through physical therapy.

## Discussion

This case highlights an unusual and severe presentation of invasive *N. meningitidis* infection complicated by a brain abscess in the absence of endocarditis. Brain abscess formation is rare in meningococcal disease and, in this context, represents an atypical focal complication that should raise concern for impaired host immune defense rather than routine invasive infection. To our knowledge, brain abscess formation in meningococcal disease in the setting of delayed diagnosis of C6 complement deficiency is rarely reported. The absence of vegetations on transesophageal echocardiography further supports hematogenous central nervous system seeding as the most likely mechanism of intracranial involvement.

A key finding in this case was the diagnosis of complement deficiency on outpatient evaluation, with low CH50 and reduced C6 levels, indicating dysfunction of both the classical and terminal complement pathways. This defect results in impaired opsonization and failure of membrane attack complex formation, which are critical for effective clearance of *N. meningitidis* [7]. Complement deficiencies are well-established risk factors for invasive meningococcal disease and are particularly associated with severe, rapidly progressive, or atypical presentations in otherwise healthy young individuals [9]. The initial normal complement studies during acute illness does not exclude complement deficiency, underscoring the importance of repeat immunologic testing in the convalescent period when suspicion remains high.

A limitation of this case is the lack of direct microbiological confirmation from the brain lesion, as biopsy or drainage was not performed. Therefore, the diagnosis of meningococcal brain abscess was based on clinical and radiographic findings, the presence of invasive *N. meningitidis* infection, and the favorable response to intravenous antimicrobial therapy.

Identifying complement deficiency carries immediate and significant clinical implications. Once diagnosed, this allows for the initiation of targeted preventive measures, including vaccination against encapsulated organisms and long-term antibiotic prophylaxis, both of which reduce the risk of recurrent invasive disease [10]. In this patient, meningococcal vaccination and monthly intramuscular penicillin were started following diagnosis in accordance with evidence-based secondary prevention strategies.

## Conclusions

Severe invasive meningococcal infection with atypical complications, such as brain abscess formation in the absence of endocarditis, should prompt an evaluation for underlying complement deficiency. Importantly, complement testing during acute illness does not exclude complement deficiency, and repeat assessment in the convalescent period is essential when clinical suspicion remains high. Early recognition of terminal complement deficiencies enables the implementation of targeted preventive strategies, including vaccination and antibiotic prophylaxis, which are critical to reducing recurrence risk and improving long-term outcomes.
